# Akzeptanz und Stand der Digitalisierung in Klinik und Praxis: Aktuelles Stimmungsbild in der deutschen Urologie

**DOI:** 10.1007/s00120-022-01889-2

**Published:** 2022-07-19

**Authors:** Angelika Borkowetz, Marianne Leitsmann, Martin Baunacke, Hendrik Borgmann, Katharina Boehm, Christer Groeben, Jan Roigas, Andreas W. Schneider, Thomas Speck, Immo Schroeder-Printzen, Susanne Zillich, Björn Volkmer, Ulrich Witzsch, Johannes Huber, Arbeitskreis Versorgungsforschung, Qualität und Ökonomie der Deutschen Gesellschaft für Urologie e. V., Arbeitskreis Informationstechnologie und Dokumentation der Deutschen Gesellschaft für Urologie e. V.

**Affiliations:** 1 Klinik und Poliklinik für Urologie, Universitätsklinikum Dresden, Technische Universität Dresden, Fetscherstraße 74, 01307 Dresden, Deutschland; 2 Klinik für Urologie, Medizinische Universität Graz, Graz, Österreich; 3 Klinik für Urologie, Universitätsmedizin Mainz, Mainz, Deutschland; 4 Klinik für Urologie, Philipps-Universität Marburg, Marburg, Deutschland; 5 Klinik für Urologie, Vivantes Klinikum Friedrichshain, Berlin, Deutschland; 6 Kooperative Belegarztpraxis für Urologie, Winsen/Luhe, Deutschland; 7 Praxis Urologie, Berlin, Deutschland; 8 Universitätsklinikum Gießen, Gießen, Deutschland; 9 Urologische und Kinderurologische Klinik, Universitätsklinikum Erlangen, Erlangen, Deutschland; 10 Klinik für Urologie, Klinikum Kassel, Kassel, Deutschland; 11 Klinik für Urologie und Kinderurologie, Krankenhaus Nordwest, Frankfurt am Main, Deutschland

**Keywords:** Digitale Medien, Digitalisierung, Elektronische Patientenakte, Videosprechstunde, Virtuelles Tumorboard, Digital media, Digitalization, Electronic patient record, Video consultation, Virtual tumor board

## Abstract

**Hintergrund:**

Die Digitalisierung der Patientendokumentation und die Einführung der elektronischen Patientenakte (ePA) stellen den klinischen Alltag vor große Herausforderungen.

**Fragestellung:**

Wir untersuchten die Akzeptanz und den Stand der Digitalisierung von Patientendaten sowie die Einführung der ePA bei den deutschen Urologen.

**Material und Methoden:**

Nach einem iterativen Entwicklungsprozess erhielten wir einen Fragebogen mit 30 Items, welcher über den Forschungsnewsletter der Deutschen Gesellschaft für Urologie e. V. versendet wurde.

**Ergebnisse:**

Insgesamt beantworteten 80 Urologen den Fragebogen (Rücklaufquote 2 %). Digitale Plattformen wie Urotube oder Researchgate wurden von 63 % der Teilnehmer verwendet. Die komplette Einführung der digitalen Patientendokumentation erfolgte bei 72 % der ambulant Tätigen und bei 54 % der in der Klinik Tätigen (*p* = 0,042). 76 % der Befragten sahen den Digitalisierungsprozess als sinnvoll an. 34 % äußerten teilweise oder starke Bedenken bzgl. einer kompletten Digitalisierung der Patientendokumentation. Nur 14 % der Teilnehmer haben eine Videosprechstunde angeboten. Als Vorteile für die ePA wurden u. a. die bessere Vernetzung des Gesundheitswesens (73 %), die Verbesserung der Diagnose, der Indikations- (41 %) und der Behandlungsqualität (48 %) sowie die Vermeidung von Fehlmedikation (70 %) gesehen.

**Schlussfolgerung:**

Die deutschen Urologen stehen der Digitalisierung der Patientendokumentation und der ePA insgesamt offen gegenüber. Insbesondere jüngere Urologen nutzen digitale Medien. Die Vorteile der Digitalisierung sind insbesondere eine Verbesserung der Behandlungsabläufe. Für eine reibungslose Einführung sind eine bereichsübergreifende Etablierung und ggf. eine Anpassung der Behandlungsprozesse notwendig.

## Abstract (en)

**Background:**

Digitalization of patient documentation and introduction of the electronic patient record (ePA) pose challenges to everyday clinical practice.

**Objectives:**

We investigated the acceptance and status of the digitalization of patient data and the introduction of the ePA among German urologists.

**Materials and methods:**

A questionnaire with 30 questions about the acceptance and status of digitalization of patient documentation and ePA was sent out via the newsletter of the German Society of Urology.

**Results:**

A total of 80 urologists participated in the survey (response rate 2%). Digital platforms such as *Urotube* or *Researchgate *are used by 63% of participants. The complete implementation of digital patient documentation was reported by 72% of respondents working in medical practice and by 54% of those working in the hospital (*p* = 0.042). While 76% see the digitalization process as reasonable, 34% expressed partial or strong concerns about the complete digitalization of patient documentation. Only 14% of the participants offer video consultations. Advantages for ePA include better networking of the healthcare system (73%), improved diagnosis, indication (41%) and treatment quality (48%), and avoidance of medication errors (70%).

**Conclusion:**

German urologists are open to the digitalization process and ePA. Especially younger urologists are using digital media. The advantages of digitalization are, in particular, an improvement in treatment processes. For a smooth introduction, a cross-departmental establishment and, if necessary, an adaptation of the treatment processes are necessary.

## Hintergrund

Insbesondere die Auswirkungen der Coronapandemie zeigen, dass Digitalisierung und elektronische Netzwerke unseren privaten und beruflichen Alltag immer mehr beeinflussen.

Die Digitalisierung hat im Gesundheitswesen einen enormen Schritt nach vorne gemacht, da die Möglichkeiten virtueller Zusammenarbeit und die Nutzung digitaler Technologien für eine weiterbestehende Versorgung eines Teils der Patienten genutzt werden konnten. Dabei werden logistische und abrechnungstechnische Prozesse schon länger digital gesteuert. Allerdings erfolgen nun zunehmend die digitale Dokumentation und Kommunikation über und mit den Patienten. Ein weiteres großes Entwicklungspotential hat die digitale Unterstützung der Behandlung von Patienten sowie die Steuerung klinischer Abläufe.

Der Krankenhausreport von 2019 mit dem Schwerpunkt der Digitalisierung im Krankenhaus zeigte einen eher schwachen Grad der Digitalisierung deutscher Krankenhäuser [1]. Das „electronic medical record adoption model“ (EMRAM) stellt ein 8‑stufiges Modell zur Messung des Digitalisierungsgrades innerhalb eines Krankenhauses dar ([1]; Tab. 1). Dabei wiesen deutsche Krankenhäuser (*n* = 167) einen EMRAM-Score von 2,3 auf. Ein großer Anteil der Krankenhäuser (38 %) wies den Stand der Stufe 0 auf. 27 % wiesen die Stufe 2 auf und fassten damit Daten aus verschiedenen Quellen in einer digitalen Patientendokumentation zusammen. Stufe 5 wurde in 18 % erreicht und nur 1 % erreichte Stufe 6 [1]. Diese Analyse zeigt, dass das deutsche Krankenhaussystem noch weit entfernt von einer kompletten Digitalisierung ist, wobei innerhalb der letzten Jahre möglicherweise der Anteil der Häuser in den Stufen 2–5 zugenommen hat [1]. Das Krankenhauszukunftsgesetz, welches im Oktober 2020 in Kraft getreten ist, soll die Digitalisierung insbesondere in den Krankenhäusern fördern. Dabei wird den Krankenhäusern ein aus Bund- und Ländern freigesetztes Fördervolumen von 4,3 Mrd. € für den Aufbau moderner Notfallkapazitäten, für die Digitalisierung und die Investition in IT-Sicherheit zur Verfügung gestellt [2].

**Table 1:** 

Stufe 0	Informationssysteme für diagnostische und versorgende Abteilungen (Radiologie, Labor) sind nicht vorhanden
Stufe 1	Informationssysteme für diagnostische und versorgende Abteilungen (Radiologie, Labor) sind vorhanden
Stufe 2	Eine elektronische Patientenakte (Patienteninformationssystem) steht für die Zusammenfassung von Daten aus verschiedenen klinischen Datenquellen zur Verfügung
Stufe 3	Digitale klinische Dokumentation und Einsatz elektronischer Verordnungen
Stufe 4	Digitale klinische Dokumentation mit Entscheidungsunterstützung
Stufe 5	Integration Bildmanagement (z. B. PACS)
Stufe 6	Klinische Dokumentation interagiert mit intelligenter klinischer Entscheidungsunterstützung und Vorhandensein eines digitalen Medikationsprozess
Stufe 7	Lückenlose elektronische Patientenakte; Data Warehouse als Basis für klinische und betriebliche Analysen

*PACS* „picture archiving and communication system“

Im stationären und ambulanten Sektor dient die Digitalisierung der Dokumentation der Vereinfachung patientenrelevanter Prozesse. Seit dem 01. Juli 2021 steht die Infrastruktur für die elektronische Patientenakte (ePA) in der Telematikinfrastruktur bereit und soll dem Patienten angeboten werden können. Die ePA ist das Kernstück der Telematikinfrastruktur, die u. a. die elektronische Arbeitsunfähigkeitsbescheinigung, den elektronischen Arztbrief, die Notfall-Informationen und Medikamente beinhaltet.

Eine wesentliche Grundlage für die Digitalisierung im Gesundheitswesen ist das E‑Health-Gesetz (§ 291 des SGB V), welches die rechtliche Grundlage für eine sichere digitale Kommunikation und Anwendung im Gesundheitswesen darstellt [3]. Ein weiterer Schwerpunkt dieses Gesetzes ist die Versorgung und Weiterentwicklung von digitalen Gesundheitsanwendungen (DiGA). DiGA sind digitale Medizinprodukte, die als sog. Apps (Applikationen) auf Rezept auf digitalen Endgeräten durch den Patient genutzt werden können. Diese werden wie Medizinprodukte mit entsprechender CE-Zertifizierung geführt. Sie sollen u. a. dazu dienen, dass die Versicherten Daten aus den DiGA auch in die ePA einstellen können. Ein weiterer wichtiger Punkt ist der Ausbau der Telemedizin, (Videosprechstunde, Erstellung der Arbeitsunfähigkeitsbescheinigung über Fernbehandlung, Ausbau der Telemedizin in Pandemiesituationen; [3–6]).

Ziel unserer Arbeit war es, die Akzeptanz und den Stand der Digitalisierung von Patientendaten und Nutzung der Digitalisierung im Krankenhaus und Praxisalltag sowie ein Stimmungsbild der ePA unter den deutschen Urologen zu untersuchen. Hierzu führten wir eine Umfrage durch, die u. a. über den Newsletter der Deutschen Gesellschaft für Urologe e. V. (DGU) versendet wurde. Dabei war abzusehen, dass eine, über allgemeine Verteiler verbreitete Umfrage, keinen repräsentativen Rücklauf erreichen würde. Ziel war es vielmehr, ein aktuelles Stimmungsbild zu erheben.

## Methoden

Unter dem Begriff Urologe sind die weiblichen, männlichen und diversen Teilnehmer inkludiert. In einem iterativen Entwicklungsprozess erarbeiteten die Autoren einen thematisch fokussierten Fragebogen mit 30 Items. Während dieses Prozesses wurden die Formulierungen optimiert und abschließend testeten 12 Freiwillige den Fragebogen. Es zeigte sich eine gute „face validity“ [7]. Im finalen Fragebogen bezogen sich 9 Fragen auf demographische Angaben und das Arbeitsumfeld, 3 Fragen auf die Nutzung digitaler Medien im klinischen Alltag, 9 Fragen auf den Stand der Digitalisierung in der Klinik oder Praxis, 6 Fragen auf die Akzeptanz der digitalen Patientendokumentation, 1 Frage auf einen möglichen Totalausfall der elektronischen Systeme sowie 2 Fragen auf die ePA. Bei 8 Fragen war eine Mehrfachantwort möglich. Der Fragebogen wurde mit dem Umfrage-Tool www.survio.com (Survio s.r.o., Brno, Tschechische Republik) im Zeitraum Juni bis September 2021 online gestellt und der Link über den monatlich erscheinenden Forschungsnewsletter der DGU an alle Mitglieder versendet. Zusätzlich nutzen wir die Verteiler der German Society of Residents in Urology (GeSRU), der Studie „Evaluation einer patientenorientierten Online-Entscheidungshilfe bei nicht metastasiertem Prostatakarzinom“ (EvEnt-PCA) sowie E‑Mail-Verteiler der Kliniken der Arbeitskreismitglieder. Insgesamt wurde der Link zum Fragebogen an ca. 5200 Personen versendet. Die Datenerhebung erfolgte anonym. Zur statistischen Auswertung wurde SPSS Statistics v27.0 (IBM, Armont, New York, USA) verwendet. Vergleiche von Subgruppen erfolgten mit dem χ^2^-Test. Zur Ermittlung von Prädiktoren für eine Beurteilung der Digitalisierung als (sehr) sinnvoll bzw. für keine bis teilweise Bedenken erfolgte eine binäre logistische Regressionsanalyse.

## Ergebnisse

Insgesamt haben 80 Urologen den Fragebogen beantwortet (Rücklaufquote 2 %). Das mittlere Alter der Teilnehmer lag bei 45 Jahren. Die in der Klinik tätigen Urologen (63 %; *n* = 50) waren signifikant jünger als die in der Praxis arbeitenden Urologen (37 %; *n* = 30; Tab. 2). 83 % der Teilnehmer waren Fachärzte für Urologie und 17 % befanden sich noch in der Facharztausbildung. 67 % der Teilnehmer waren > 10 Jahre als Urologe tätig. 58 % der Teilnehmer kamen aus den alten Bundesländern. 62 % waren im großstädtischen Einzugsgebiet (> 100.000 Einwohner) tätig.

**Table 2:** 

Variable	Gesamtkohorte	In der Klinik tätige Ärzte (*n* = 50)	In der Praxis tätige Ärzte (*n* = 30)	*p*
*Alter in Jahren (MW [±* *SD])*	45 (± 11,8)	40 (± 10,4)	53,5 (± 8,9)	< 0,001
*Geschlecht (n [%])*				
Frauen	18 (23)	13 (26)	5 (17)	–
Männer	61 (77)	36 (74)	25 (83)	0,310
*Einzugsgebiet (n [%])*				
Ländlich (< 20.000 Einwohner)	6 (8)	0	6 (20)	–
Kleinstädtisch	21 (26)	11 (22)	10 (33)	–
Großstädtisch	53 (66)	39 (78)	14 (47)	–
*Nutzung digitaler Medien (Mehrfachantwort möglich; n [%])*				
Soziale Medien	42 (53)	30 (60)	12 (40)	0,083
Gesundheits-Apps	77 (96)	49 (82)	28 (93)	0,287
Primärliteratur und Leitlinien	16 (20)	13 (26)	3 (10)	0,083
Plattformen (z. B. *Researchgate*)	50 (63)	37 (74)	13 (16)	0,006
Berufsbegleitende Fortbildungsmöglichkeiten	67 (84)	38 (76)	18 (60)	0,072
Es werden keine genutzt	0	0	0	–
*Durchführung Videosprechstunde*	10 (12,5)	9 (18)	1 (3)	0,055
*Durchführung eines Web-basierten Tumorboards*	31 (39)	22 (44)	9 (30)	0,213
*Digitalisierung als (sehr) sinnvoll betrachtet*	62 (78)	45 (90)	17 (57)	0,115
*Stand der Einführung der digitalen Patientendokumentation (n [%])*				
Gar nicht	2 (3)	0	2 (7)	–
Teilweise eingeführt, aber vorrangig Dokumentation auf Papier	7 (9)	5 (10)	2 (7)	–
Teilweise eingeführt, Dokumentation vorranging digital	22 (27)	18 (36)	4 (14)	–
Komplett digitalisiert	49 (61)	27 (54)	22 (72)	0,042
*Bedenken zur Digitalisierung der Patientendokumentation (n [%])*				
Keine Bedenken	54 (68)	35 (70)	19 (64)	–
Teilweise Bedenken	24 (30)	14 (28)	10 (33)	–
Starke Bedenken	2 (2)	1 (2)	1 (3)	0,805
*Schwierigkeiten bei der Umsetzung (n [%])*				
Keine Schwierigkeiten	10 (13)	2 (4)	8 (27)	–
Vorübergehend mäßige Schwierigkeiten, die überwindbar sind	31 (39)	20 (40)	11 (37)	–
Vorübergehend mäßige Schwierigkeiten, die aber schnell überwindbar waren	14 (18)	9 (18)	5 (17)	0,037
Schwierigkeiten, die nicht überwindbar waren	0	0	0	–

*MW* Mittelwert, *SD* Standardabweichung

### Nutzung digitaler Medien und Nutzung der digitalen Sprechstunden

Die Nutzung digitaler Medien bei in der Klinik und Praxis tätigen Urologen ist in Tab. 2 dargestellt. Soziale Medien (*p* = 0,01) und Plattformen (*p* < 0,01) wurden häufiger von jüngeren Urologen genutzt (< 42 Jahre = medianes Alter). Gesundheits-Apps wurden häufiger von Urologen < 50 Jahren (freigewählt) verwendet (*p* = 0,01). Nur 14 % der Befragten haben eine Videosprechstunde angeboten. Hier zeigte sich kein Hinweis darauf, dass dieses Angebot von jüngeren Urologen (< 42 Jahren oder < 50 Jahren) häufiger verwendet wurde (*p* = 0,6 bzw. 0,2). 39 % der Teilnehmer nutzten ein webbasiertes, virtuelles Tumorboard. Hier zeigte sich ebenso kein Hinweis, dass dieses Angebot von jüngeren Urologen (< 42 Jahren oder < 50 Jahren) häufiger verwendet wurde (*p* = 1,0 bzw. 0,9).

### Digitalisierung der Patientendokumentation in Klinik und Praxis

Der Stand der Digitalisierung in der Klinik und Praxis ist in Tab. 2 abgebildet. Die komplette Einführung der digitalen Patientendokumentation erfolgte bei 72 % (22/30) ambulant tätiger Teilnehmer und bei 54 % (27/50) in der Klinik tätigen Teilnehmern (*p* = 0,042).

Bei den Teilnehmern, die schon mit einer kompletten digitalen Dokumentation in der Praxis/Klinik arbeiteten, wurden bei 44 % die digitale Patientendokumentation vor > 4 Jahren, bei 13 % vor 3–4 Jahren, bei 29 % vor 1–2 Jahren und bei 14 % < 1 Jahr eingeführt. Dabei wurde die digitale Patientendokumentation in 71 % stufenweise und bei 20 % an einem Stichtag eingeführt.

Folgende Maßnahmen wurden bei der Implementierung der digitalen Patientendokumentation ergriffen: regelmäßige Teamsitzungen mit Vertretern aller Berufsgruppen (51 %), ausführliche Analyse und ggf. Anpassung aller klinik-/praxisrelevanter Abläufe im Vorfeld (49 %), Ausbau anderer digitaler Medien bzw. Programme (z. B. elektronisches Managementhandbuch; 34 %) und Schulung von *Key-Usern* in den Abteilungen/Bereichen, die weitere Mitarbeiter unterstützten und anleiteten (56 %). 18 % gaben an, dass die Mitarbeiter im Aufbau der digitalen Patientendokumentation nicht einbezogen wurden. Bei 31 % der Teilnehmer wurde eine ausführliche Schulungsmöglichkeit der Anwender durchgeführt. Bei 40 % der Teilnehmer gab es eine ausführliche Beratung und einen umfassenden Support bei der Implementierung. 49 % der Teilnehmer gaben einen teilweisen Support durch die IT-Abteilung oder durch eine externe Firma an. Bei 11 % der Teilnehmer war die Klinik oder Praxis vollkommen auf sich alleine gestellt. Bezüglich des Datenschutzes gaben 78 % der Teilnehmer eine Beratungsmöglichkeit bzw. Überprüfung der Einhaltung des Datenschutzes an. Für den Fall eines Totalausfalls des Systems gaben 45 % der Teilnehmer an, dass sie sich mit diesem Thema noch nicht beschäftigt haben. Weitere 24 % gaben an, dass für diesen Fall keine Konzepte vorlagen. 16 % der Teilnehmer gaben an, dass diese Notfallpläne in ausgedruckter Form in allen Bereichen eingesehen werden können.

**Figure 1:**
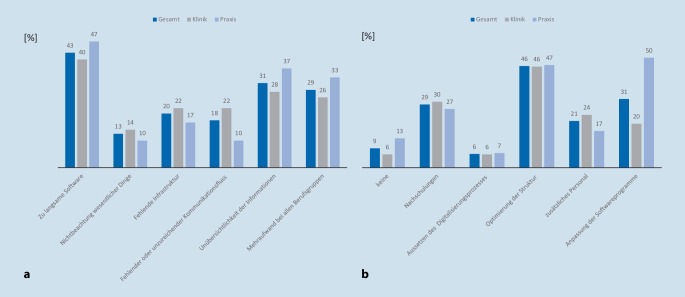

Die Teilnehmer, bei denen die Digitalisierung teilweise bzw. komplett umgesetzt wurde, gaben die in Abb. 1a dargestellten Schwierigkeiten an. Dabei wurden folgende Maßnahmen zur Optimierung der Prozesse und Qualität nach der Implementierung durchgeführt: Nachschulungen (29 %), Aussetzung des Digitalisierungsprozesses (6 %), Optimierung der Struktur der elektronischen Akte (46 %), Einsatz von zusätzlichem Personal (z. B. Scankraft; 21 %), sowie Anschaffung oder Anpassung von weiterer Software (31 %; Abb. 1b).

### Akzeptanz der digitalen Patientendokumentation in Praxis und Klinik

Als Ziele der Digitalisierung der Patientendokumentation sahen die Teilnehmer v. a. die allzeitige Verfügbarkeit der vollständigen Patientendokumentation (88 %), die allgemeine Zugänglichkeit von Informationen über Abläufe (63 %), eine leichtere Vernetzung der IT-Systeme (66 %), die Unterstützung von Diagnose und Therapie z. B. durch „Clinical-decision-support“-Systeme (40 %) und die Sicherstellung des Datenschutzes (30 %).

Die Digitalisierung in der Patientenversorgung bewerteten 78 % der Teilnehmer als sehr sinnvoll oder sinnvoll. 34 % der Teilnehmer äußerten teilweise oder starke Bedenken bzgl. einer kompletten Digitalisierung der Patientendokumentation. Die übrigen 66 % hatten keine Bedenken.

**Figure 2:**
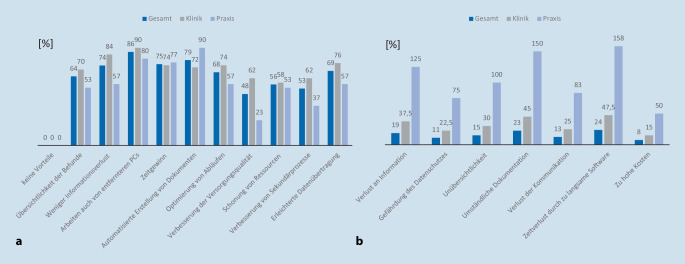

Die Teilnehmer sahen eine Reihe an Vorteilen der Digitalisierung der Dokumentation in Praxis und Klinik (Abb. 2a). Die bestehenden Bedenken bezogen sich bei 24 % der Teilnehmer auf den Zeitverlust durch zu langsame Software und bei 23 % auf eine zu umständliche Dokumentation einfacher Befunde (Abb. 2b).

In der binär-logistischen Regressionsanalyse waren folgende Parameter mit der Einschätzung der Digitalisierung als sehr sinnvoll oder sinnvoll assoziiert: Alter < 42 Jahren (Odds Ratio [OR]: 14,2; 95 %-Konfidenzintervall [KI]: 2,9–67,5; *p* < 0,001), Arbeit in der Klinik (OR: 6,9; 95 %-KI: 2,1–22,3; *p* = 0,001), großstädtisches Einzugsgebiet (OR: 3,5; 95 %-KI: 1,1–11,1; *p* = 0,029) und das Nicht-Vorhandensein von Bedenken (OR: 6,9; 95 %-KI: 2,2–21,6; *p* < 0,001). Unabhängiger Prädiktor für die Einschätzung als sehr sinnvoll oder sinnvoll war das Alter < 42 Jahre (OR: 14,5; 95 %-KI: 2,12–99,07 *p* = 0,006) und das Nicht-Vorhandensein von Bedenken (OR: 12,24; 95 %-KI: 2,64–56,64 *p* = 0,001). Urologen aus den neuen Bundesländern hatten dabei mehr Bedenken (keine Bedenken vs. teilweise oder starke Bedenken) bei der Einführung der Digitalisierung in Praxis und Klinik als Urologen aus den neuen Bundesländern (51 % vs. 20 %; *p* = 0,003).

## Stimmungsbild zur ePA im Rahmen der Telematikinfrastruktur

**Figure 3:**
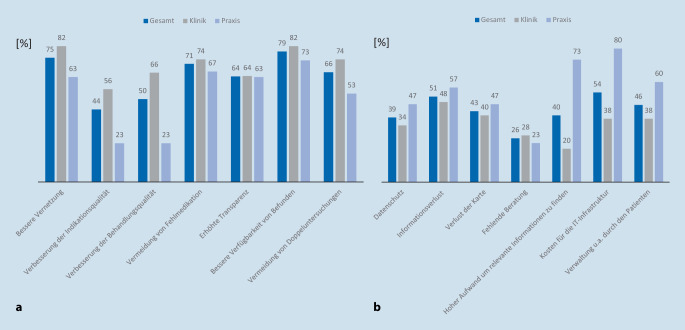

Die Abb. 3a, b zeigen die von den Befragten empfundenen Vorteile und Bedenken gegenüber der ePA. Eine verbesserte Vernetzung im Gesundheitswesen, der Informationsqualität und Behandlungsqualität durch die ePA sahen v. a. die in der Klinik arbeitenden Urologen. Die in der Praxis tätigen Urologen äußerten insbesondere Bedenken bzgl. des hohen Aufwands relevante Informationen zu gewinnen, bzgl. der Kosten für die Infrastruktur und bzgl. der Verwaltung durch den Patienten.

## Diskussion

Die Ergebnisse unserer Umfrage zeigen, dass die Digitalisierung der Patientendokumentation bei 28 % der Teilnehmer teilweise und bei 59 % komplett in Deutschland erfolgte. Hierbei zeigt sich, dass die komplette Digitalisierung der Patientendokumentation häufiger in der Praxis als in der Klinik eingeführt worden war. In der Mehrzahl erfolgte der Übergang bereits vor über 3 Jahren. Dies ist besonders wichtig im Kontext der aktuellen Umstellung auf die ePA seit 01.07.2021 im ambulanten Bereich und seit 2022 auch im stationären Sektor [5]. Nur 7 % der Teilnehmer gaben an, dass eine Digitalisierung der Patientendokumentation nicht vorgesehen ist.

Ein wichtiger Bestandteil der Einführung der digitalen Patientendokumentation sind gemeinsame Teamsitzungen zur Integration aller Mitarbeiter in den Digitalisierungsprozess. Die Analyse aller relevanten Abläufe ist ein essentieller Schritt im Digitalisierungsprozess. Für die Akzeptanz der digitalen Patientendokumentation sind entsprechende Schulungen der Mitarbeiter und die Etablierung sog. *Key-User *notwendig, die in den einzelnen Bereichen zur Unterstützung anderer Mitarbeiter dienen sollen. Daher ist die enge Anbindung an eine IT-Abteilung bzw. externe Anbieter wichtig. Diese spielt insbesondere beim Management von Totalausfällen eine wesentliche Rolle. Daher ist es erstaunlich, dass nur ein geringer Anteil der Teilnehmer weiß, wie sie sich bei einem Totalausfall zu verhalten haben.

Bei der Implementierung der Digitalisierung gaben 45 % der Teilnehmer eine zu langsame Software an. Diese Problematik ist sowohl in der Praxis, als auch in der Klinik gleichstark evident. Auch die Unübersichtlichkeit der Informationen und ein zeitlicher Mehraufwand wurden in bis zu 30 % der Fälle genannt. Daher waren im Nachhinein v. a. Nachschulungen und die Optimierung der Struktur der digitalen Patientendokumentation sowie die Anschaffung und Anpassung weiterer Software notwendig. Betriebswirtschaftliche Chancen durch die Digitalisierung im Krankenhaus und Praxis werden v. a. durch die Steigerung der Behandlungsqualität, Steigerung der Prozesseffizienz, Reduzierung der Kosten, einer Optimierung der Auslastung sowie Reduktion der Verweildauer bei Vertretern der Krankenhäuser und Pflegeeinrichtungen angesehen [1]. Lediglich 11 % der Teilnehmer gaben Bedenken bzgl. des Datenschutzes an. Letzteres ist v. a. deswegen erstaunlich, da doch ein Drittel der Krankenhäuser Opfer von Hackerangriffen wurden [8]. Dies begründet die Notwendigkeit eines ausreichenden Sicherheitssystems durch entsprechende *Firewalls* und Datenverschlüsselungen. 2020 führte die KBV eine Umfrage im Bereich der ambulanten Versorgung im Rahmen des Praxisbarometers mit Schwerpunkt Digitalisierung durch [9]. Hier sahen die Ärzte und Psychotherapeuten durch die Einführung der ePA v. a. einen Nutzen für die Organisation ihrer Praxis [9]. Verbesserungen wurden in der Umfrage des Praxisbarometers bei etwa einem Viertel in der Diagnose- und Indikationsqualität, sowie in der Wirtschaftlichkeit der Patientenversorgung gesehen. Nur ein Fünftel der Befragten sehen eine Verbesserung der Behandlungsqualität durch die ePA in der Umfrage des Praxisbarometers [9]. In unserer Umfrage dachte die Hälfte der Befragten, dass sich mit der ePA die Behandlungsqualität verbessert, insbesondere aus der Sicht der in der Klinik tätigen Urologen. Zu beachten ist, dass 37 % der Befragten im Praxisbarometer durch die ePA eine Verschlechterung der Arzt-Patient-Beziehung sehen. Fast ein Drittel sieht noch eine Verschlechterung der Verwaltung und des Organisationsmanagements. Starke Bedenken haben die Befragten bzgl. der technischen Umsetzung und deren Fehleranfälligkeit. Hier existieren starke Hemmnisse aus Sicht der Befragten. 81 % sehen Sicherheitslücken in der EDV [9]. Dies steht im Widerspruch zu unserer Umfrage, bei der nur 11 % Sicherheitslücken sahen. Fast ebenso viele befürchteten ein ungünstiges Kosten-Nutzen-Verhältnis und 82 % die Fehleranfälligkeit der IT. Allerdings sahen etwa die Hälfte der Befragten des Praxisbarometers eine Verbesserung in der Kommunikation mit dem stationären Sektor und ärztlichen Kollegen im ambulanten Bereich [9].

Die Verzögerungen der Einführung des elektronischen Rezepts (eRezept) oder Arbeitsunfähigkeitsbescheinig (eAU) spiegeln Schwierigkeiten der Implementierung im deutschen Gesundheitssystems wider. So wurde die endgültige Umsetzung des eRezepts zu Beginn des Jahres 2022 in nochmals eine verlängerte, noch unbefristete Testphase geschickt, um bestehende Probleme zu evaluieren [10]. Für die eAU ist endgültige Implementierung nun für Mitte 2023 vorgesehen [10].

In der von uns durchgeführten Umfrage konnte außerdem gezeigt werden, dass v. a. die jüngeren Teilnehmer sehr viel häufiger auf soziale Medien zur Vernetzung und digitale Plattformen zum Wissenserwerb setzten. Die am häufigsten genutzten Sozialen Medien in der Urologie sind *Youtube, Facebook, Twitter* und *Instagram *[11–13]. Rivas *et al.* zeigten, dass soziale Medien einen wichtigen Stellenwert in der Aneignung von Fachwissen besitzen [12]. 99 % der Befragten nutzten Soziale Medien, wobei *YouTube* und *LinkedIn* die häufigsten genutzten Plattformen zur Vernetzung darstellten. *YouTube* wurde v. a. zur Aneignung von chirurgischen Techniken genutzt. *Facebook* oder *Twitter* rangierten auf dem dritten Platz. Weiter hinten platzierten sich Angebote über Fachgesellschaften, Kongresse oder urologische Fachzeitschriften [12]. Dies ist kritisch zu betrachten, da es insbesondere über *YouTube* oder andere soziale Medien verbreitete Informationen möglicherweise an entsprechender Evidenz mangeln. Gibt man bei *YouTube* den Begriff „Urology“ ein, so werden über 50.000 Videos angeboten [14].

Nur 14 % der Teilnehmer gaben an, dass sie eine Videosprechstunde anbieten. Dies ist noch ein zu geringer Anteil, wenn man bedenkt, dass das Angebot der Videosprechstunde ein Bestandteil der ePA bzw. von einigen DiGA darstellen wird [15].

Der eHealth-Report 2021 zeigte, dass insbesondere für die Onlinesprechstunde bzw. Zweitmeinung einen starken Aufwärtstrend in den letzten beiden Jahren erfolgte [16]. Die Kommunikation zwischen den Akteuren des Gesundheitswesens (Leistungserbringer und Kostenträger) soll verstärkt auf die Telematikinfrastruktur zurückgreifen und die digitale Kommunikation zwischen Patienten und Arzt in Form von Videosprechstunden weiter ausgebaut werden. Hier spielt die Entwicklung der Videokommunikations- und *Messengerdienste* eine herausragende Rolle [17]. Im Zuge der Coronapandemie wurde bei den deutschen Ärzten und in den Psychotherapiepraxen die Nutzung der Videosprechstunde massiv erhöht. Unterstützt wurde dies durch die Aufhebung der gelten Restriktionen durch die KBV und den GKV-Spitzenverband im zweiten Quartal 2020 [18]. Web-basierte Tumorboards wurden hingegen bei 38 % der Teilnehmer durchgeführt.

### Stärken und Einschränkungen

Es bestehen eine Reihe von Limitationen unserer Untersuchung. Neben den grundsätzlichen Problemen einer Umfrage wie z. B. den Antworten im Sinne der sozialen Erwünschtheit, ist der geringe Rücklauf von ca. 2 % die stärkste Einschränkung. Zudem besteht ein starker Selektionsbias aufgrund der Verlinkung der Umfrage im digitalen Newsletter der Fachgesellschaft. Die erhaltene Stichprobe ist damit sehr wahrscheinlich nicht repräsentativ. Wie oben ausgeführt, entkräfteten aber vergleichbare Ergebnisse aus der Literatur das Selektionsbias. Trotz dieser methodischen Einschränkungen erscheint das erhaltene Stimmungsbild zur Digitalisierung in der deutschen Urologie wertvoll.

## Fazit für die Praxis

- Insbesondere die jüngeren Urologen nutzen die digitalen Plattformen für den Wissenserwerb oder in der Diagnose- und Therapieentscheidung.
- Die deutschen Urologen stehen der Digitalisierung der Patientendokumentation und der elektronischen Patientenakte (ePA) sowohl im stationären als auch im ambulanten Sektor offen gegenüber.
- Die Vorteile von Digitalisierung und ePA sind insbesondere eine Verbesserung der Behandlungsabläufe. Für eine reibungslose Einführung sind eine bereichsübergreifende Etablierung und Analyse sowie ggf. eine Anpassung der Behandlungsprozesse notwendig.
- Der Ausbau der Digitalisierung wird weiter voranschreiten. Neben den nun ergriffenen Maßnahmen werden in Zukunft insbesondere „Clinical-decision-support“-Systeme für die Erstellung von Diagnose- und Behandlungsabläufe eine wichtige Rolle spielen.
- Die durch die Digitalisierung und die ePA gewonnen gesundheits- und krankheitsbezogenen Daten können dabei helfen, Behandlungs- und Therapieabläufe besser zu definieren.
- Zukünftige Potenziale der Digitalisierung liegen im Ausbau der Datenverwaltung und der Verbesserung der Interaktion zwischen Patienten und Leistungserbringern.
- Hierfür ist es notwendig die gesetzlichen Rahmenbedingungen der Datenakquise der wissenschaftlichen Nutzung auch umzusetzen.
